# Ultrasonographic Predictors of Difficult Laparoscopic Cholecystectomy

**DOI:** 10.7759/cureus.93870

**Published:** 2025-10-05

**Authors:** Ibrahim Asim, Ahmed Taymour Algahiny, Omar HeshamEldin Abouelella, Malak Mohamed Refaat Shehab El Din, Omar Samir Mohamed Megahed Saleh Elmitwalli, Mohamed Bakr Elnagar, Haya Khaled Ali Abdulla AlKhalifa, Ahmed Mostafa Abdalla Mohamed, Hosam Alazazzi

**Affiliations:** 1 Radiology, University Hospitals of Leicester NHS Trust, Leicester, GBR; 2 Bioengineering, Imperial College London, London, GBR; 3 Pharmacology, University of Oxford, Oxford, GBR; 4 Medicine, Royal College of Surgeons in Ireland, Muharraq, BHR; 5 Surgery, University Hospitals of Leicester NHS Trust, Leicester, GBR; 6 Internal Medicine, Newgiza University, Cairo, EGY; 7 Internal Medicine, Salford Royal NHS Foundation Trust, Manchester, GBR; 8 Internal Medicine, Leeds Teaching Hospitals NHS Trust, Leeds, GBR; 9 Internal Medicine, Hamad Medical Corporation, Doha, QAT; 10 Gastroenterology, Wrightington, Wigan and Leigh NHS Foundation Trust, Wigan, GBR

**Keywords:** common bile duct, difficult laparoscopic cholecystectomy, gall bladder, gallbladder wall thickness, gallstones, laparoscopic cholecystectomy, pericholecystic fluid, scoring systems, ultrasound, ultrasound predictors

## Abstract

Laparoscopic cholecystectomy (LC) is the gold-standard treatment for gallstone disease, but some procedures become technically challenging, resulting in longer operative times, higher conversion rates, and increased complications. Accurate preoperative prediction is essential for safe surgical planning. This review summarizes current evidence on ultrasonographic predictors of difficult LC (DLC) and evaluates validated ultrasound-based scoring systems for preoperative risk stratification. Ultrasound remains the first-line modality for gallbladder assessment and provides several key predictors of surgical difficulty: gallbladder wall thickness (GBWT) consistently demonstrates the strongest independent association with DLC, correlating with conversion and postoperative complications. Pericholecystic fluid, though less frequent, is highly specific for severe inflammation and complex cases. Gallstone impaction at the neck or Hartmann’s pouch is a stronger predictor than stone multiplicity. Abnormal gallbladder size, whether contracted or distended, complicates dissection, while common bile duct dilatation serves as a secondary predictor. Several scoring systems combine these sonographic features with clinical variables such as age, sex, obesity, and prior inflammation, helping achieve stronger predictive accuracy.

## Introduction and background

Laparoscopic cholecystectomy (LC) is widely recognized as the gold-standard treatment for gallstone disease [1]. Compared to open cholecystectomy (OC), it offers many advantages, including less postoperative pain, shorter hospital stays, quicker recovery, and better cosmetic results [2]. However, when LC becomes technically difficult, it is associated with increased operative time, greater intraoperative blood loss, longer hospital stays, higher complication and conversion rates, elevated treatment costs, and increased mortality [3]. Current data suggest that between 1% and 13% of all LCs require conversion to open surgery, depending on the scenario [4,5]. Even though difficult cases are reported in up to 26% of surgeries, there is still no universally agreed-upon definition of what makes an LC “difficult,” which complicates preoperative planning and risk reduction [3].

At present, no single preoperative scoring system can reliably predict surgical complexity, leaving surgeons to rely heavily on experience and intraoperative judgment. Significant risk factors for conversion include a BMI over 30 kg/m², male sex, history of acute cholecystitis or pancreatitis, prior upper abdominal surgery, and gallbladder wall thickness greater than 3 mm [6]. Additional studies highlight age over 60, comorbidities, prior abdominal operations, contracted or thickened gallbladders, and impacted stones as further predictors of difficult LC [4,7,8].

Ultrasound is generally the first-line imaging modality for gallbladder assessment due to its non-invasive nature, wide availability, and cost-effectiveness. Unlike CT or magnetic resonance cholangiopancreatography (MRCP), which are typically reserved for complex or equivocal cases, ultrasound can be performed quickly at the bedside without exposing patients to ionizing radiation, while providing highly relevant clinical information. A variety of sonographic features - such as a thickened gallbladder wall, stones impacted in the neck, dilated common bile duct, pericholecystic fluid, and a shrunken or contracted gallbladder - have been associated with increased surgical difficulty [9-11]. Researchers have sought to combine these findings into scoring systems aimed at predicting which cases are likely to be more challenging; for example, Siddiqui et al. [12] developed a model demonstrating promising predictive value for conversion risk.

Predicting difficult LC preoperatively is clinically valuable beyond academic interest. Identification of high-risk cases allows for careful allocation of operative time and resources, ensures the involvement of experienced surgeons, and facilitates informed preoperative counselling for patients. By emphasizing ultrasound as the primary assessment tool, clinicians can harness its availability, cost-effectiveness, and predictive capacity to optimize surgical planning and outcomes. This literature review aims to summarise current evidence on ultrasonographic predictors that have been statistically linked to difficult LC and to evaluate the various ultrasound-based scoring systems developed to anticipate these challenging cases.

## Review

Methods

A comprehensive literature review was conducted using reliable databases, including PubMed, Scopus, and Google Scholar, to identify studies evaluating ultrasonographic predictors of DLC. Search terms included “difficult laparoscopic cholecystectomy”, “ultrasound predictors”, “gallbladder wall thickness”, “gallstones”, “gall bladder diameter”, “common bile duct diameter”, and “scoring system”. Inclusion criteria comprised original studies, published in English, that assessed preoperative ultrasonographic or clinico-radiological predictors of DLC in adult patients only. Exclusion criteria included case reports, editorials, and conference abstracts. Following screening of titles, abstracts, and full texts, the literature included spanned observational studies, retrospective/prospective cohorts, and validation studies of ultrasound-based scoring systems. Data were extracted on study design, population characteristics, ultrasonographic parameters, scoring systems, and predictive performance metrics.

Surgical assessment

A DLC lacks a universally accepted definition but is generally characterized by prolonged dissection, conversion to open surgery, or the need to manage complex anatomical or pathological conditions. Contributing factors include severe inflammation, impacted stones, fibrotic (scleroatrophic) gallbladders, abscesses, or bile duct injury (BDI) [13,14]. An example is when having a “frozen Calot’s triangle” caused by chronic inflammation or fibrosis obscures the biliary anatomy and increases the risk of BDI [14,15]. Most studies define procedural difficulty as an operative time exceeding 90 minutes or a conversion to open surgery due to safety concerns [15]. Reported incidence ranges from 10% to 26% in the general population [13-15]. Conversion to open surgery is not a failure but rather a safety measure taken when laparoscopic dissection cannot be performed adequately. Surgeons may convert when key structures cannot be clearly identified, when excessive bleeding compromises visibility, or when severe adhesions prevent safe progress. The decision reflects an attempt to minimize the risk of catastrophic complications such as BDI, vascular injury, or prolonged operative time.

Effective preoperative risk stratification is essential for optimizing surgical outcomes in LC. It enables tailored operative planning, ensures the availability of experienced surgeons and advanced equipment, and facilitates thorough patient counselling on treatment options/outcomes [16]. Identifying difficult cases in advance reduces the likelihood of unplanned conversion and supports consideration of safer alternatives such as subtotal/open cholecystectomy [17]. Multiple validated scoring systems that incorporate clinical, biochemical, and ultrasonographic parameters aid in anticipating procedural difficulty, particularly in cases of inflammation or fibrosis. Failure to predict surgical complexity has been associated with increased rates of BDI and operative delays. A multicenter study of more than 20,000 cases found that DLCs accounted for nearly 70% of conversions and 80% of BDIs [4]. The contrast in outcomes between routine and DLC highlights the value of accurate preoperative risk assessment, as shown in Table 1 [3,7-10]. Difficult procedures are associated with increased operative time, higher conversion and complication rates, prolonged hospital stays, and slightly elevated mortality. These associations support the clinical relevance of predictive models in identifying high-risk cases. Stratification using such data supports resource allocation, referral decisions, and surgical triage in emergency settings.

**Table 1:** Variations in surgical parameters between routine and difficult LC. LC: Laparoscopic cholecystectomy

Parameter	Routine LC	Difficult LC
Operative time	30–60 min	>90–120 min
Conversion to open	2–5%	10–30%
Bile duct injury	0.3–0.5%	Up to 2%+
Hospital stays	1 day	3–5 days
Mortality	<0.1%	Slightly increased

Ultrasound predictors

Ultrasound remains the first-line modality for assessing gallbladder pathology and provides key predictors of operative difficulty. The following subsections investigate the relevant sonographic features of cholecystitis.

Gallbladder Wall Thickness (GBWT)

GBWT is among the most extensively studied ultrasonographic predictors of DLC. Pathologically, a thick wall is indicative of inflammation, edema, or fibrosis, factors that obscure dissection planes, increase the risk of complications, prolong the procedure, and may ultimately result in conversion to an open procedure.

A prospective cohort study of 1,089 patients divided GBWT into three categories: less than 3 mm, 3-7 mm, and greater than 7 mm. They noted an increase in intraoperative events and complexity of procedures as the thickness increased. Importantly, regardless of adjusting for symptom duration, GBWT remained an independent predictor, with an odds ratio (OR) of 2.1 for the thickest group [18]. These findings align with earlier data from a retrospective study of 874 patients, where conversion to open surgery rose from 3.1% in those with 1-2 mm GBWT to 16.8% in those ≥7 mm. The same study also found that patients with thicker walls experienced significantly longer hospital stays and higher postoperative complication rates [19].

A regional Indian study of 350 patients followed this pattern, with an alarming 100% rate of complications in moderate-to-severe GBWT cases, in addition to significantly longer surgery and hospital stay durations [9]. Similarly, a Sudanese study of 110 patients concluded that GBWT readings over 3 mm indicated longer operation times and difficult intraoperative cases, though it did not quantify conversion or complication rates [20]. Further reinforcing these findings, a 700-patient study demonstrated that increasing GBWT was significantly associated with longer operative times, higher intraoperative difficulty, and a greater likelihood of conversion to open cholecystectomy [21].

In terms of diagnostic performance, a study found that GBWT >4 mm had a sensitivity of 81.8% and specificity of 97.2% in predicting DLC [22]. Another prospective analysis reported that GBWT >4 mm achieved 50% sensitivity, 93.9% specificity, and an AUC of 0.749, reinforcing its predictive value for technical difficulty [23]. In addition to its predictive accuracy, a study of 69 patients showed that a GBWT of 3 mm or more was significantly associated with conversion to open cholecystectomy, where conversion occurred only in those with thickened walls, while those with normal GBWT had a 0% conversion rate [24].

Although cut-offs vary, literature constantly shows that GBWT is a strong and consistent predictor of DLC in diverse health-care environments in different regions, as summarised in Table 2. However, heterogeneity in threshold values and measurement techniques across studies may affect reproducibility in routine clinical practice.

**Table 2:** Comparison of GBWT cut-off values and associated key findings with statistical significance. GBWT: Gallbladder wall thickness

Study (Year)	Sample Size	GBWT Cut-Off(s)	Key Findings	OR/P-value/Sensitivity/Specificity
Kokoroskos et al. (2020) [18]	1,089	>7 mm	Increasing intraoperative events and complexity with thicker walls	OR: 2.1
Raman et al. (2012) [19]	874	>7 mm	Thicker walls linked to longer stay & higher complications	P<0.001
Khan et al. (2023) [9]	350	>6 mm	100% complication rate; longer surgery & hospital stay	P<0.0001
Salah et al. (2021) [20]	110	>7 mm	Longer operation times, more difficult intraoperative cases	P<0.05
Balbaloglu et al. (2023) [21]	700	>3 mm	Longer operative times, higher difficulty, greater conversion risk	Sensitivity: 80.79%, Specificity: 68.58%
Stanisic et al. (2020) [22]	369	>4 mm	Strong predictive value for DLC	Sensitivity: 81.8%, Specificity: 97.2%
Darwish et al. (2022) [23]	50	>4 mm	Predictive accuracy of GBWT confirmed	Sensitivity: 50%, Specificity: 93.9%
Mishra et al. (2023) [24]	69	>3 mm	Conversion only in thickened walls; 0% conversion in normal GBWT	Sensitivity: 75.6%, Specificity: 67.16%

Pericholecystic Fluid (PF)

PF occurs as a halo of anechoic or echogenic fluid around the gallbladder and signifies acute inflammation and, in some cases, microperforation. Though it occurs less frequently than gallbladder wall thickening, its high diagnostic specificity makes it an important confirmatory finding when surgical complications are expected.

In a prospective observational study of 100 patients, PF showed 100% specificity and 100% positive predictive value (PPV) for DLC, though with a sensitivity of just 20.8% [22]. While these values are striking, the low sample size and lack of multivariate adjustment reduce confidence in their standalone predictive power. A larger study of 180 patients found that fluid presence increased the likelihood of DLC to 80.95% (compared to 23.27% without fluid) and conversion to open surgery to 33.33% (vs. 1.88%). These corresponded to a sensitivity of 70%, specificity of 91.76%, PPV of 33.33%, and NPV of 98.11% [2]. This was also demonstrated in a Sudanese population, where PF was implicated in increased operative difficulty, often in association with GBWT and impacted calculi, thus increasing its predictive value [20].

To further validate these findings, Chhaparia et al. [25] demonstrated that PF, gallbladder wall thickening, and impacted stones were among the most reliable ultrasonographic predictors of difficult laparoscopic cholecystectomy. Their analysis confirmed that patients with these features had significantly higher rates of intraoperative difficulty, conversion to open surgery, and prolonged operative time. This combination-based approach aligns more closely with real-world clinical assessments, though its predictive accuracy was not validated prospectively. Similarly, a recent multicenter retrospective study by Kim et al. [26] confirmed that PF, alongside gallbladder wall thickening, was a significant independent ultrasonographic predictor of gangrenous cholecystitis, reinforcing its diagnostic value in severe disease. In a wider radiologic context, its presence was also linked with patients requiring emergent surgery.

In light of this, the finding of PF is one with high specificity. While the lack of it does not rule out complications with certainty, its presence in combination with GBWT and other such structural findings ought to be of concern and warrant careful surgical preparation. However, variations in reporting in the literature, reliance on qualitative assessments, and variations in criteria for diagnosis make it difficult to establish it as a valid independent predictor. The available data are compelling but often derived from relatively small, single-center cohorts, underscoring the need for large, multi-center prospective studies to validate their predictive utility and standardize reporting practices.

Gallstone Location and Multiplicity 

Gallstones are frequently identified on sonography in patients undergoing laparoscopic cholecystectomy, but not all stone characteristics carry the same prognostic weight. Evidence consistently demonstrates that stone location, particularly impaction, provides greater predictive value for operative difficulty than stone multiplicity.

Siddiqui et al. [12], in a prospective study of 300 patients, reported that impacted gallstones were significantly associated with DLC (p < 0.05). Conversely, the presence of multiple stones (>1) showed no predictive significance (p = 0.74). In their scoring system, impaction was given substantial weight, confirming its stronger predictive value compared to multiplicity. Similarly, Randhawa et al. [17] included impacted stones in their predictive scoring system, finding a significant positive correlation with operative complexity.

This finding has been echoed in focused studies by Singh et al. [27], who demonstrated that 43.5% of patients with impacted stones experienced prolonged operative times and more difficult dissections, reinforcing the role of impaction as a critical sonographic predictor. Supporting this, Ahmed [28] observed that stones impacted in Hartmann’s pouch were strongly associated with distorted anatomy, difficulty in grasping the gallbladder, and higher rates of conversion to open surgery.

Taken together, these studies establish that stone impaction and location are consistently more reliable predictors of surgical difficulty than stone multiplicity, which offers limited prognostic value. Thus, when stratifying patients preoperatively, sonographic evidence of an impacted stone should be regarded as a high-risk marker, whereas the number of stones alone should not alter surgical planning.

*Gallbladder Diameter* 

Although their definitions varied amongst studies, gallbladder contraction and distension have both been identified as indicators of intraoperative complexity. A gallbladder transverse diameter ≥ 5 cm was regarded by Siddiqui et al. [12] as a significant risk factor and earned two points in their ultrasound-based difficulty scoring system. According to their results, this marker could predict DLC with a sensitivity of 80.7% and a specificity of 91.7%. Its clinical value is further supported by its high diagnostic accuracy.

Teja et al. [29] noted that the diameter of the gallbladder is an important predictor in multivariate prediction models, with higher diameters being linked with longer operative times. However, knowledge about the existence of threshold effects is limited, mainly because of the insufficiency of analysis with respect to size strata. It's interesting to note that O'Leary et al. [30] analyzed the opposite spectrum, contracted gallbladders, and discovered that 16% of patients with shrunken gallbladders needed open surgery. With a statistically significant p-value of 0.005, their multivariate analysis revealed gallbladder contraction as an independent indicator.

Common Bile Duct (CBD) Diameter

Despite the variability of findings, the dilation of CBD has been studied as a secondary indicator of technical complexity. Siddiqui et al. [12] classified a CBD diameter of more than 6 mm as two points and found a significant relationship between the variable and the incidence of difficult laparoscopic cholecystectomy (p < 0.05). Along with GB size and impaction, CBD dilatation was considered an equal contributor in their scoring system. O'Leary et al., however, found that, although CBD dilatation was significant in univariate analysis (p = 0.0416), it was not significant in the multivariate model. This implies that, when other sonographic parameters are controlled, its predictive value decreases [30].

Further evidence by Teja et al. [29] demonstrated that the average CBD diameter in converted cases was 6.45 mm, with a p-value of 0.001. Although the statistical strength is clear, the study's predictive independence was limited because of the variation in the operating surgeon and only inclusion of only elective cases. Moreover, Teja et al. [29] found that, while CBD diameter showed an association with LC difficulty in univariate analysis, it was not an independent predictor in multivariate analysis; instead, GBWT and stone impaction emerged as stronger indicators of operative complexity.

Discussion

LC remains the definitive surgical management of cholecystitis, especially when complicated by gallstones [31]. A DLC can be characterised by the technical challenges involved in resection due to underlying pathophysiology - including inflammatory changes, structural alteration in surgical anatomy, and rising incidence of complication,s including risks of injury to surrounding structures [32]. The ability to predict the difficulty of the procedure allows surgeons to plan ahead in terms of surgical strategy and risk management while helping triage patients, leading to better outcomes and recovery in both the short and long term [33]. Operationally, a DLC can be measured by prolonged operative time (>90 minutes), conversion rates to open surgery, or intraoperative complications such as bile duct injury. Other important measures include length of hospital stay and mortality [34].

Ultrasound is an easily accessible and widely used first-line radiological investigation that helps to diagnose cases for LC. Our review has highlighted the key sonographic features that correlate to DLC, including the GBWT, PF, gallstone location, and multiplicity, as well as the GB and CBD diameters [11]. GBWT consistently emerges as the most reliable predictor of DLC across numerous studies. There is a positive correlation showing that, with an increase in wall thickness, there is a corresponding increase in the difficulty of the LC, including associated complications [18]. Although studies vary in the cut-off values for marked increase in difficulty, it can be established that, in the majority of studies, a cut-off of a wall thickness ≥3 mm is correlated with increased operative time, higher conversion rates, and more postoperative complications [9]. 

PF occurs less frequently than GBWT; however, when it does occur, it is strongly predictive of a DLC. It is closely associated with more complex diseases where there is severe inflammation, such as gangrenous or perforated cholecystitis, often a good indicator for those who need emergent surgery [26]. In terms of gallstone location and multiplicity, location turns out to be the more reliable prognostic factor, with significant data showing that impaction at the gallbladder neck and Hartmann's pouch is predictive of DLC [35]. GB diameter was also raised as a predictor in a few scoring systems. However, there is no well-studied cut-off with no standardisation in measurement and correlation with surgical difficulty. Additionally, interestingly, both contracted and over distended GB sizes were shown to complicate surgery [36]. Finally, CBD diameter turns out to be a good predictor in the context of other markers, but again with no well-studied cut-off, it remains a secondary predictor of DLC [37].

It is notable that reported statistics for certain outcomes in laparoscopic cholecystectomy vary in range, highlighting heterogeneity between studies. Such variability may be influenced by multiple factors, including geographic location, differences in healthcare infrastructure, surgical expertise, hospital volume, and case mix. Another important varying factor is the inconsistent methodology in ultrasonographic assessment. Standardized protocols, such as measurements after a uniform fasting period (six to eight hours) and specifying the imaging plane (transverse vs. axial), could improve reproducibility and clinical utility. Despite variability in techniques, thresholds, and operator dependency, the consistent patterns across different populations suggest that incorporating these parameters into standardised scoring systems could enhance preoperative risk stratification and surgical planning.

At the moment, there are a few ultrasound-based scoring systems that have been developed to predict the likelihood of a DLC, each using different combinations of sonographic and clinical variables. One of the earliest and widely cited models is the Randhawa scoring system [17], which was developed based on patient history, clinical examination, and ultrasound findings, with a total of 15 points, with four attributed to sonographic findings. It demonstrated high accuracy in predicting both easy and difficult cases, with success rates of 88.8% and 92%, respectively.

Gupta et al. validated the scoring system of Randhawa, using the same criteria for defining a DLC, and also reported high sensitivity and specificity of 95.74% and 73.68%, respectively [16]. Both models include GBWT, PF collection, and impacted stones as key ultrasonographic predictors, with GBWT being the most significant sonographic factor. Further studies have also verified the utility of the Randhawa scoring system [38,39].

Tongyoo et al. [40] modified the Randhawa scoring model by incorporating radiologically contracted gallbladder into the scoring. They also broadened the criterion of previous hospitalisation for acute cholecystitis to include prior inflammation and procedures such as cholangitis and endoscopic retrograde cholangiopancreatography (ERCP) [40]. These modifications improved the model’s applicability in predicting the difficulty of elective LC while maintaining its ease of use for preoperative planning.

Siddiqui et al. [12] developed a scoring system based solely on sonographic features, including features that were not in the Randhawa model such as transverse gallbladder diameter, CBD diameter, number of stones, and enlarged liver. This system demonstrated strong diagnostic performance, correctly identifying 66 of 83 difficult cases and 199 of 217 uncomplicated cases, yielding a sensitivity of 80.7% and a specificity of 91.7%. The overall predictive accuracy was approximately 79% for difficult procedures and 93% for straightforward cases [12].

Nassar et al. [41] also developed and validated a pre-operative risk score to predict the difficulty of LC using the Nassar intra-operative difficulty grading scale. Analysis of two large patient cohorts (CholeS study with 8,755 cases and a single-surgeon reference cohort of 4,089 cases) identified eight independent predictors of difficult surgery: age ≥40 years, male gender, higher ASA classification, diagnosis of cholecystitis or CBD stones, thick-walled gallbladder, CBD dilation, pre-operative ERCP, and non-elective admission. A scoring system based on these factors showed good predictive accuracy, effectively stratifying patients into low, medium, and high-risk categories for surgical difficulty. Table 3 summarizes these different scoring systems.

**Table 3:** Summary of the different scoring systems. ROC: Receiver operating characteristics; AUROC: Area under the receiver operating characteristic curve

Scoring System	Key Variables	Ultrasound Predictors Included	Other Predictors	Sample Size	Diagnostic Performance
Randhawa et al. [17]	15-point system (clinical, history, US)	GBWT, pericholecystic fluid, impacted stone	Age, sex, previous admissions, palpable GB, BMI, abdominal scar	228	Easy cases predicted: 88.8%; Difficult cases predicted: 92%
Gupta et al. [16]	Validation of Randhawa	Same as Randhawa	Same as Randhawa	210	Sensitivity 95.7%; Specificity 73.7%
Tongyoo et al. [40]	Modified Randhawa model	Same as Randhawa + radiologically contracted GB	Broadened prior hospitalization criteria (incl. cholangitis, ERCP)	567	Better correlation and a higher ROC curve than the Randhawa model
Siddiqui et al. [12]	Sonography-only score	GBWT, pericholecystic fluid, impacted stone, transverse GB diameter, CBD diameter, number of stones, enlarged liver	None (US-only model)	300	Sensitivity 80.7%; Specificity 91.7%; Accuracy ~79% (difficult), 93% (easy)
Nassar et al. [41]	Validated risk score derived from a new scoring system	GBWT, CBD dilation	Age ≥40, male sex, ASA class, cholecystitis or CBD stones, pre-op ERCP, non-elective admission	CholeS (8,755) + single-surgeon (4,089)	Stratified risk into low, medium, high categories; good predictive accuracy AUROC: 0.789 (95% CI 0.773–0.806)

While ultrasound remains indispensable in the preoperative assessment of gallbladder disease, its role in predicting the ease or difficulty of LC does have inherent limitations. Ultrasound is highly operator-dependent, with findings only as clear as the skill of the scanner, and image quality itself may be degraded by factors such as obesity or excessive bowel gas. More critically, though, several determinants of intraoperative difficulty cannot be adequately assessed with ultrasound alone. For example, the degree of subserosal fibrosis, dense adhesions from previous inflammation or surgery, and anatomical distortion of Calot’s triangle are poorly visualised on standard sonography, yet these are among the most influential factors contributing to surgical complexity, operative time, and conversion risk [42]. Furthermore, ultrasound cannot differentiate between reversible inflammatory edema and irreversible fibrotic change. Adhesive disease secondary to pancreatitis or prior peritonitis, as well as vascular anomalies within Calot’s region, also remain largely undetectable. Some of these limitations can be addressed with the use of further imaging modalities such as computed tomography (CT), which offers improved visualisation of pericholecystic inflammation, and magnetic resonance cholangiopancreatography (MRCP), which provides superior resolution of biliary anatomy and is particularly valuable when Mirizzi syndrome or bile duct injury is suspected [43,44].

However, it is important to highlight, though, that, beyond imaging modalities, there are various other clinical features in the literature that should also be taken into account in the assessment of LC difficulty and when designing a comprehensive scoring system - male gender and age over 60 years are linked to more fibrotic or inflamed gallbladders, complicating dissection [45]. Obesity increases operative challenges due to reduced visibility and excess intra-abdominal fat [15]. A history of acute cholecystitis, pancreatitis, or previous upper abdominal surgery raises the risk of adhesions and anatomical distortion, making surgery more complex [46]. Delayed presentation (symptoms lasting >72 hours) and elevated inflammatory markers such as white blood cell count or CRP also correlate with higher conversion rates [47]. These clinical predictors are valuable for preoperative risk stratification, especially when ultrasound findings are equivocal. Another limitation of this review is the absence of a pooled statistical analysis, which restricts the ability to draw definitive conclusions regarding comparative effectiveness. Future research should aim to conduct high-quality meta-analyses or systematic reviews that quantitatively synthesize data across multiple studies, allowing for more robust estimation of predictive accuracy, identification of consistent risk factors, and potential development of validated scoring systems to guide preoperative planning.

## Conclusions

LC is the standard treatment for cholecystitis, particularly when gallstones are involved. DLC is defined by prolonged operative time, higher conversion rates to open surgery, complications such as bile duct injury, and extended recovery. Accurate prediction of DLC improves surgical planning and patient outcomes. Ultrasound, as a first-line tool, identifies key sonographic features to this effect: primarily, GBWT is the most consistently correlated marker of DLC. Pericholecystic fluid, though less frequent, has high specificity for DLC as a marker of inflammation, along with gallstone impaction, indicating technical complexity. Secondary features, such as contracted/distended GB size and CBD dilation, also suggest DLC; however, specific cut-offs and techniques of evaluating these parameters vary - further quantitative studies can verify and incorporate all these features into ultrasound-based risk stratification systems. Additionally, clinical factors such as male sex, increased age, obesity, prior inflammation, surgery, delayed presentation, and elevated inflammatory markers further inform DLC risk and could also be included in the comprehensive assessment and design of scoring systems. Looking ahead, novel modalities such as elastography may offer a more objective assessment of gallbladder wall stiffness, while AI-based image analysis holds promise for automated, reproducible prediction models. These approaches could enhance accuracy and consistency, paving the way for robust and clinically useful DLC prediction tools.
